# A High-Strength Strain Sensor Based on a Reshaped Micro-Air-Cavity

**DOI:** 10.3390/s20164530

**Published:** 2020-08-13

**Authors:** Yanping Chen, Junxian Luo, Shen Liu, Mengqiang Zou, Shengzhen Lu, Yong Yang, Changrui Liao, Yiping Wang

**Affiliations:** 1 Guangdong and Hong Kong Joint Research Centre for Optical Fibre Sensors, College of Physics and Optoelectronic Engineering, Shenzhen University, Shenzhen 518060, China; chenyanping2019@email.szu.edu.cn (Y.C.); 1810285012@email.szu.edu.cn (J.L.); zmq@guet.edu.cn (M.Z.); lushengzhen2019@email.szu.edu.cn (S.L.); y.yang28@aston.ac.uk (Y.Y.); cliao@szu.edu.cn (C.L.); ypwang@szu.edu.cn (Y.W.); 2 Guangdong Laboratory of Artificial Intelligence and Digital Economy (SZ), Shenzhen University, Shenzhen 518060, China; 3 Key Laboratory of Optoelectronic Devices and Systems of Ministry of Education and Guangdong Province, Shenzhen University, Shenzhen 518060, China

**Keywords:** Fabry–Perot interferometer, strain sensor, fiber optics sensors, optical sensing and sensors

## Abstract

We demonstrate a high-strength strain sensor based on a micro-air-cavity reshaped through repeating arc discharge. The strain sensor has a micro-scale cavity, approximate plane reflection, and large wall thickness, contributing to a broad free spectrum range ~36 nm at 1555 nm, high fringe contrast ~38 dB, and super-high mechanical robustness, respectively. A sensitivity of ~2.39 pm/με and a large measurement range of 0 to 9800 με are achieved for this strain sensor. The strain sensor has a high strength, e.g., the tensile strain applied the sensor is up to 10,000 με until the tested the single-mode fiber is broken into two sections. In addition, it exhibited low thermal sensitivity of less than 1.0 pm/°C reducing the cross-sensitivity between tensile strain and temperature.

## 1. Introduction

A variety of fiber-optic sensors for strain sensing applications have been investigated, such as those based on Fiber Bragg Gratings (FBGs) [1,2], helical long-period fiber grating [3], Fabry–Perot interferometers (FPIs) [4,5,6,7], Mach–Zehnder interferometers (MZIs) [8,9,10], and hybrid structures [11,12,13]. Typically, the strain sensors based on fiber-optic FPIs have been widely used in areas of biomedicine, automotive industries, and environmental monitoring [14], due to their outstanding advantages of high sensitivity, compact size, and simple structure for potentially low-cost fabrication [5]. Recently, for measuring strain, the FPIs based on an in-fiber air cavity attracted a great deal of attention due to their excellent advantages, e.g., small thermal expansion coefficient; and a variety of methods for in-fiber air cavities fabrication have been reported and demonstrated in last few years. For example, a novel and simple technique for fabricating air-cavity-based FPI were reported in Ref. [5] and Ref. [15] that only a commercial refractive index matching liquid and a common fusion splicer were employed in the whole fabrication process. This technique was further utilized to fabricate a unique rectangular-air bubble-based strain sensor with a high sensitivity of 43.0 pm/με [6], unfortunately, the sensor has a low mechanical strength due to the ultra-thin wall thickness of the air-cavities of about 1 μm [9]. Liao et al. [16] reported an in-grating bubble sensor, with strain tested ranges from 0 to 1000 με, fabricated by femtosecond (fs) laser ablation together with a fusion-splicing technique. Cibula et al. [17] proposed to fabricate an air cavity inside a single-mode fiber using the combination techniques both etching and splicing, and the strain response of the device was measured from −2500 to 2500 με. Moreover, the in-fiber FPIs for measuring tensile strain could be also fabricated by employing special optical fiber (SOF) structures, for instance, hollow-core photonic crystal fibers (PCFs) [18,19,20], Hollow-Core Fiber (HCF) [21,22] and so on. However, almost all the strain sensors mentioned above have a relatively small measurement range that far lower than the limited deformability of silica fiber [23]. Besides, the strain sensors with SOF have the problem of lacking SOFs components, such as splitters, circulators, switches, etc., the incompatibility of most SOFs with telecommunications optical fibers make SOF strain sensors complex and impractical [24].

In this paper, we experimentally demonstrate a high strength strain sensor based on the FPI with a micro-air-cavity, which is fabricated using a single-mode fiber (SMF, Corning Inc., SMF28) and can be reshaped by repeating arc discharge. The cavity length can suffer an extremely small change via control of the discharge time and power, resulting in the dip resonance wavelength of the FPI readjusted. Thus, it can be designed to operate at optical telecommunication wavelengths, making the strain sensor simpler and more practical. As an example, an in-fiber FPI, with a dip wavelength of 1550.06 nm and an extinction ratio of ~37.6 dB, was created by reshaping the cavity length of a micro-air-cavity into about 26 μm; where the controlled accuracy of the dip resonance wavelength is better than 60 pm. Moreover, such an FPI can be developed into a strain sensor, which has a strain sensitivity of ~2.39 pm/με and a large measurement range from 0 to 9800 με, almost reaching to the limited deformability of silica fiber. It has the potential application of concrete buildings, which often behave elastically and are often subjected to a strain of less than 2000 με under normal loads [23]. Besides, the strain sensor has a low thermal sensitivity of less than 1.0 pm/°C. Such an optical fiber strain sensor has the advantages of low-temperature cross-sensitivity and high strength, and it is suitable for large strain measurement. 

## 2. FPI Dip Wavelength Customization

Herein, we have successfully customized a target dip wavelength for the FPI through fine adjusting the cavity length, which was realized by reshaping the micro-air-cavity through arc discharge, thus fine adjusting the dip wavelength. Assume the target dip wavelength for the FPI is 1550 nm, and the detailed process of adjusting the dip wavelength to 1550 nm for sample-1 is as an example and demonstrated as follows.

**Figure 1:**
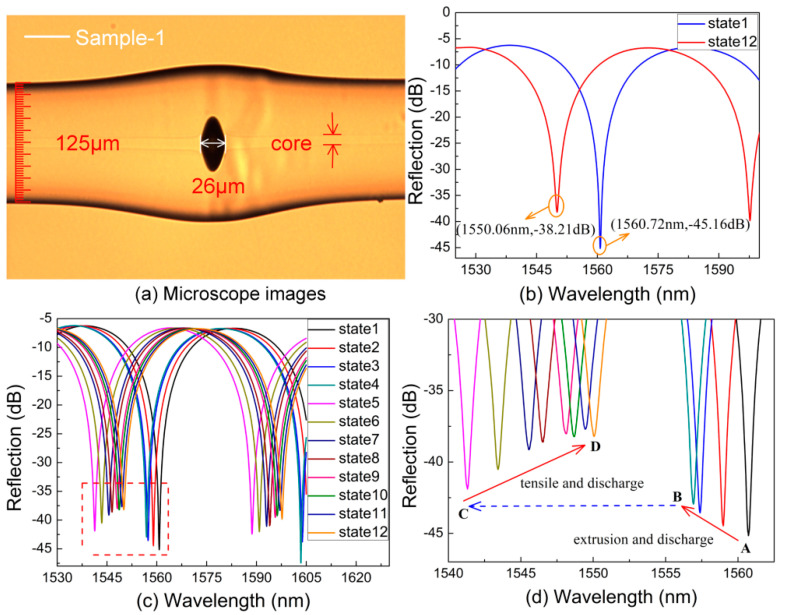
(**a**) Microscope images of the in-fiber Fabry–Perot interferometers (FPI) with a cavity length of 26 μm. (**b**) The reflection original spectrum and the final spectrum. (**c**) The dip wavelength of reflection spectra at the same arc discharge states (state 1–12). (**d**) Enlarged the view of the dotted box.

The detailed processes of micro-air-cavity fabrication are well described in previous studies [5,6]. In summary, an in-fiber FPI with a micro-air-cavity length is obtained as sample-1, and the corresponding optical microscope image of the sample-1 after being reshaped is shown in Figure 1a. We use a measurement device consists of a 3-dB fiber coupler, a super-continuum source (SC source, YSL Photonics), and an optical spectrum analyzer (OSA, YOKOGAWA AQ6370C) with a limit resolution of 0.02 nm. The reflection original spectrum (state 1) and the final spectrum (state 12), i.e., the before and after being reshaped spectra with the dip wavelength of 1560.72 nm and 1550.06 nm, respectively, are shown in Figure 1b. From Figure 1b, we observed the dip wavelength of sample-1 moved close to 1550 nm clearly after a series of reshaping operations, which is the result of the cavity length decreased. Comparing to the target resonance wavelength 1550 nm, the deviation is only about 60 pm, which also can be reduced by precisely controlling the discharge time and power.

Figure 1c shows the evolution spectra of the in-fiber FPI based on the micro-air-cavity in the reshaping process, which adjusts the dip wavelength of the FPI’s interference fringe by means of extruding and repeating local electrical arc discharge. The dip wavelength of reflection spectra at the same arc discharge states (state 1–12) in different times are more clearly described in Figure 1d, which is an enlarged view of the dotted box in Figure 1c. As shown in Figure 1d, the dip wavelength 1560.72 nm of the initial air cavity with a loss of −45.16 dB is marked as A. To achieve the target dip wavelength of 1550 nm, we firstly extruded the in-fiber bubble along the axial direction by moving the two fiber ends with a distance of 10 μm, resulting in two walls of the in-fiber bubbles being subjected to axial stress. The next step is to repeat local electrical arc discharge, which is to melt the fiber slightly and then cool down at room temperature, releasing part of the axial stress of the bubble wall and thus compressing the cavity length of the in-fiber micro-air-cavity. The power and time of each discharge are precisely controlled, and the bubble is extruded only once before the first discharge. In the process of controlling the dip wavelength drifting from A to B, the discharge parameters are set as standard-50 bit and 200 ms, and the spectrum after several times of discharging is shown as state-4 in Figure 1c,d. A dip wavelength of 1556.92 nm of the FPI with a loss of −42.95 dB is achieved, i.e., the point marked with B, which is much closer to the target wavelength 1550 nm.

To demonstrate the reshaping process more visually and conveniently, we increased the discharge parameters to be standard-20 bit and 500 ms, and then continue reshaping the bubble. Since these discharge parameters are larger than needed, we achieved an over-adjusted dip wavelength of 1541.32 nm, i.e., the point marked as C in Figure 1d, which is to the left of the target dip wavelength 1550 nm. To adjust the dip wavelength toward to 1550 nm, two holders stretch the sample-1 along the axial direction by moving in opposite directions ~5 μm. The discharge parameters are set to be standard—50 bit and 200 ms. Repeating the discharge process above, we achieved a series of reflection spectra with different dips of wavelength during the repeating reshapes, corresponding to reshape states (state C to D), shown in Figure 1d. After several repeated fine-adjusting processes, we achieved a dip wavelength of 1550.06 nm, i.e., the point marked D, which is very close to the target dip wavelength 1550 nm, and the deviation is only about 60 pm. In addition, after the steps above, the optical fiber micro-cavity has a considerable thickness, which will result in high robustness.

## 3. Strain Sensing and Discussion

**Figure 2:**
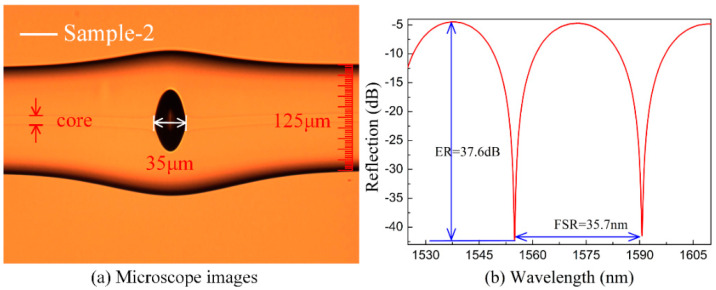
(**a**) Microscope images of the in-fiber FPI with a cavity length of ~35 μm. (**b**) The corresponding reflection spectrum of the FPI, free spectral range (FSR) ~35.7 nm, ER ~37.6 dB.

As shown in Figure 2a, another micro-air-cavity with a cavity length of about 35 μm is denoted as sample-2, which is created by using the fabrication method mentioned above. The corresponding reflection spectrum of sample-2 was recorded with wavelength ranges from 1525 to 1610 nm, as shown in Figure 2b. The free spectral range (FSR) of sample-2 is ~35.7 nm, the extinction ratio was ~37.6 dB, and a dip of the resonant wavelength was 1555.12 nm.

**Figure 3:**
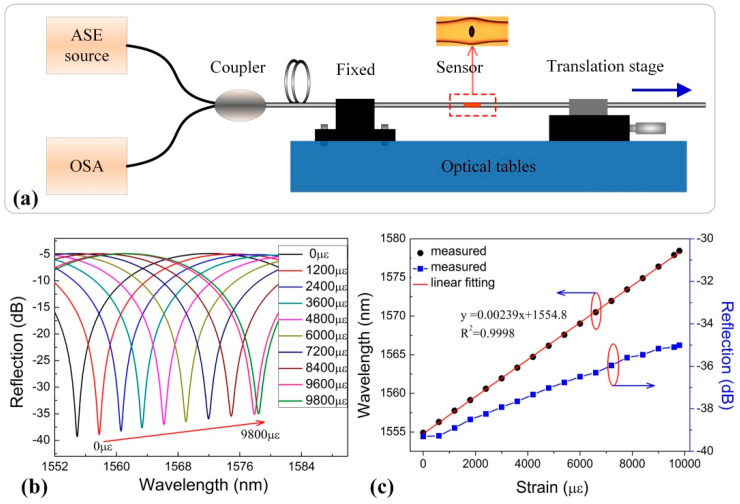
(**a**) Experimental setup for strain measurement. (**b**) Wavelength shift of interference fringe around 1555 nm as a function of tensile strain applied to the air-cavity-based FPI sample. (**c**) Reflection spectrum evolution of the air-bubble-based FPI sample while the tensile strain increasing from 0 to 9800 με.

To evaluate the strain response of these devices, an experiment was set up to apply tensile strain to sample-2, as shown in Figure 3a. One end of the FPI was fixed, and the other end was firmly attached to a translation stage with a resolution of 10 μm. The total length of the stretched fiber, including the SMF and in-fiber FPI, is 100 mm. The dip wavelength shift of the reflection fringe around ~1555 nm was measured while the tensile strain is increased from 0 to 9800 με with an increment step of 200 με. Figure 3b shows the evolution of the FPI reflection spectrum as the tensile strain increased at room temperature, and the corresponding strain sensitivity is ~2.39 pm/με. The dip wavelength shifts toward longer wavelengths as the axial strain increases, due to the cavity length of the in-fiber FPI increasing. In addition, the loss of dip changes a little, less than 0.03% of the reflection of a flat SMF end surface. As shown in Figure 3c, a strain sensitivity of 2.39 pm/με is obtained in the proposed FPI by linear fitting of the dip wavelength change.

**Figure 4:**
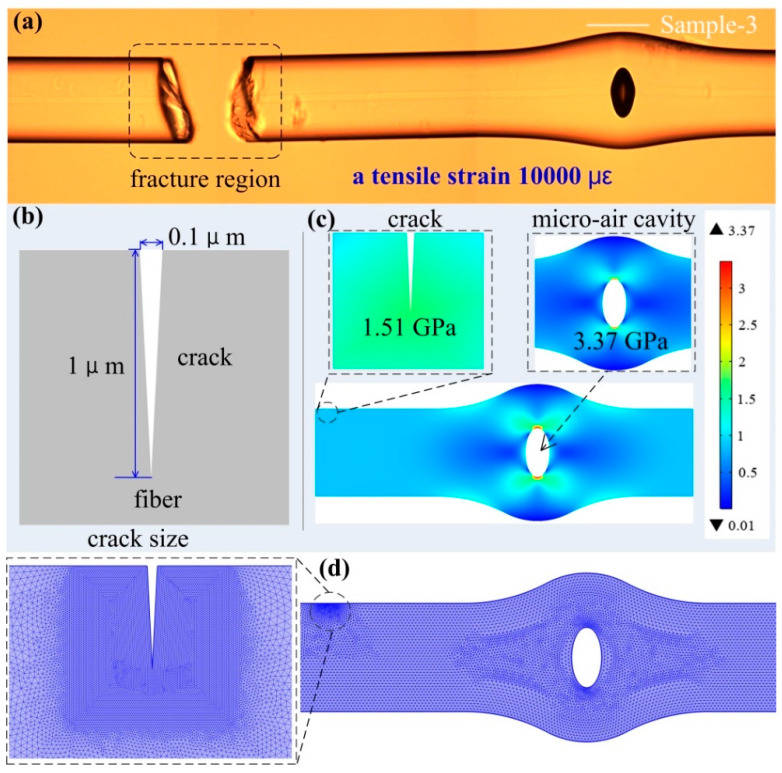
(**a**) Microscope images of the in-fiber FPI with fracture surface appears in the fiber. (**b**) Geometric model of crack in-fiber. (**c**) Two-dimensional stress contours while the axial strain of 10,000 με applied to the model. (**d**) Mesh of the in-fiber FPI model.

The tensile strength of the FPI was investigated by continuously increases tensile strain to a sample-3 until the tested sample breaks into two sections. In the process of applying 10,000 με axial strain to the FPI, the main part of the SMF was torn while the bubble part was intact, it is suggested that the strain sensor with the FPI based on a reshaped bubble is robustness. The microscope image of the broken SMF with the in-fiber FPI based on the micro-air-cavity is shown in Figure 4a.

To investigate the stress distribution and the deformation of the in-fiber FPI based on the micro-air-cavity and crack under an applied tensile strain in the tensile strength test, we established a simulation model using commercial finite element analysis software, and the results are shown in Figure 4b,c. We employed standard parameters in the simulations for silica [6], including a silica density of 2700 kg/m^3^, Young’s modulus of 73 GPa, and the Poisson’s ratio of 0.17. The simulation model is established including a crack in the fiber surface and a micro-air cavity with geometric dimension parameters achieved from the experiment. Figure 4b illustrates the length and depth of the crack model, corresponding to 0.1 and 1 μm, respectively. When the axial strain of 10,000 με is applied to the model, the simulated 2D stress contours of the air cavity and crack in-fiber are shown in Figure 4c. We observed that the value of stress distribution on the air cavity and the crack in-fiber is nearly uniform. The values of stress distribution are 1.51 GPa and 3.37 GPa corresponding to the crack in-fiber and the micro-air cavity, respectively, which shows that the air cavity can stand much more stress than the crack in the fiber surface. It is worth noting that we adopted the mesh of free triangular in the whole meshing, and the free triangular was finer around the crack tip, which makes the mesh around the crack tip much denser, as shown in Figure 4d.

**Figure 5:**
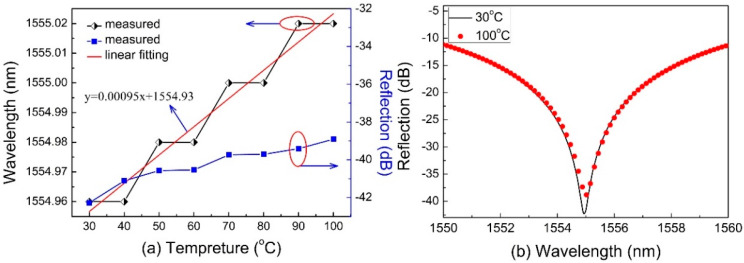
(**a**) Linear relationship between the wavelength shift of the interference dip at ~1555 nm and ambient temperature. (**b**) The reflection spectra of FPI at 30 °C and 100 °C.

The temperature response was also investigated by placing the FPI sample-2 with a cavity length of 35 μm in a column oven and then raising the temperature from 30 °C to 100 °C with a step of 10 °C, remaining for 10 min at each temperature rise step. Figure 5a shows the wavelength shift and loss change of the dip wavelength at ~1555 nm as the temperature increases, which is clearly observed to be a redshift with an approximately linear relation temperature sensitivity of only ~0.95 pm/°C. Obviously, the obtained temperature sensitivity of the FPI sample-2 is relatively low when compared with other fiber-optic temperature sensors, such as FBG (~9 pm/°C) [25], long-period fiber grating (LPFG) 114.2 (nm/°C) [3]. Besides the temperature sensitivity of ~0.95 pm/°C is at the same level as the reported air cavity fabricated by splicing (~1 pm/°C) in Ref. [4,5,26]. The reflection spectra of FPI at 30 °C and 100 °C are shown in Figure 5b. Thus, the temperature-induced strain measurement error will be smaller than 0.4 με/°C if no temperature compensation is done.

## 4. Conclusions

In conclusion, we experimentally demonstrate an in-fiber FPI based on a micro-air-cavity for high-strength strain sensitivity measurement. We propose to precisely alter the resonant wavelength by direct reshaping the micro-air-cavity. The dip resonance wavelength can be precisely controlled by reshaping the micro-air-cavity, which is realized through either tension or extrusion by the fiber holders along with repeating arc discharge. After the reshaping process, the cavity wall thickness of the strain sensor increases. Such a thick-wall micro-air-cavity sensor exposes high robustness, large strain response range, and low-temperature cross-sensitivity. Under the strain of 0 to 9800 με, the strain sensitivity is ~2.39 pm/με, while the temperature cross-sensitivity is smaller than 0.4 με/°C. Since the tensile strain applied to the strain sensor is up to 10,000 με, the sensor has a feature of high strength. Moreover, the stress distribution of the sensor is simulated by finite element software, and the simulation results show that the thick-wall micro-air-cavity structure has high robustness. The strain sensor has advantages of low cross-sensitivity between tensile strain and temperature, easy fabrication process, high strength, and low cost. It is promising for high-strain sensing in practical harsh environments.
